# Safety Concern between Autologous Fat Graft, Mesenchymal Stem Cell and Osteosarcoma Recurrence

**DOI:** 10.1371/journal.pone.0010999

**Published:** 2010-06-08

**Authors:** Pierre Perrot, Julie Rousseau, Anne-Laure Bouffaut, Françoise Rédini, Elisabeth Cassagnau, Frédéric Deschaseaux, Marie-Françoise Heymann, Dominique Heymann, Franck Duteille, Valérie Trichet, François Gouin

**Affiliations:** 1 INSERM, U957, Nantes, France; 2 Université de Nantes, Nantes Atlantique Universités, Laboratoire de Physiopathologie de la Résorption Osseuse et Thérapie des Tumeurs Osseuses Primitives, EA3822, Nantes, France; 3 Centre Hospitalier Universitaire, Service de Chirurgie Plastique et des Brûlés, Nantes, France; 4 Centre Hospitalier Universitaire, Service d'Anatomie Pathologique, Nantes, France; 5 Etablissement Français du Sang Centre-Atlantique, EA3855, Tours, France; 6 Centre Hospitalier Universitaire, Service d'Orthopédie-Traumatologie, Pôle Ostéo-articulaire, Nantes, France; University of Oxford, United Kingdom

## Abstract

**Background:**

Osteosarcoma is the most common malignant primary bone tumour in young adult treated by neo adjuvant chemotherapy, surgical tumor removal and adjuvant multidrug chemotherapy. For correction of soft tissue defect consecutive to surgery and/or tumor treatment, autologous fat graft has been proposed in plastic and reconstructive surgery.

**Principal Findings:**

We report here a case of a late local recurrence of osteosarcoma which occurred 13 years after the initial pathology and 18 months after a lipofilling procedure. Because such recurrence was highly unexpected, we investigated the possible relationship of tumor growth with fat injections and with mesenchymal stem/stromal cell like cells which are largely found in fatty tissue. Results obtained in osteosarcoma pre-clinical models show that fat grafts or progenitor cells promoted tumor growth.

**Significance:**

These observations and results raise the question of whether autologous fat grafting is a safe reconstructive procedure in a known post neoplasic context.

## Introduction

In plastic and reconstructive surgery, autologous fat grafting enables soft tissue augmentation and is increasingly used for cosmetic indications but also for correction of defect following tumor treatment. This procedure proposed by Coleman was applied first for facial remodelling and more recently for breast augmentation [1], [2]. This lipofilling procedure is usually considered as a safe procedure and does not require a permit application. Indeed it consists of autologous tissue injection in a site of defect, directly after harvesting without *ex vivo* expansion.

The only safety concern which was predicted in 1987 by the American Society of Plastic and Reconstructive Surgeons was that fat grafting may compromise breast cancer detection by inducing microcalcifications. In fact a recent clinical study has demonstrated that previous breast augmentation leads to difficulties for breast cancer detection and for tumor management and reconstruction procedure [3]. Concerning breast reconstruction after mastectomy, fat injection seems to be accepted, but it remains controversial to treat cosmetic sequelae after conservative breast surgical treatment [4], [5]. Indeed one safety question concerns the potential risk of recurrence caused by fat injection side effect, but this question remains unanswered for breast cancer as relapse incidence is already high for certain groups of patients.

In contrast, late local recurrence is an unexpected event after complete remission of an osteosarcoma. Only 5% of patients with recurrent disease have local recurrence and such event occurs at a median time ranging from 6 to 28 months as reported by Ferrari *et al.* [6]. Additionally these authors have observed a disease free survival of 46% stable from the 8^th^ to 12^th^ year. In another study, Meyers *et al.* have found the latest recurrence at the 9^th^ year [7]. Bielack *et al.* have only reported 2 cases of late relapse over the 204 patients followed during 10 years; both were distant relapses with no local recurrence [8]. In fact, late local recurrence (after 15 to 20 years) has only been reported for low grade parosteal osteosarcoma subtype [9].

Here we report an unexpected local osteosarcoma relapse which occurred at the exact site of autologous fat grafts in a female patient who did not present any predictive factor of local recurrence. Previous experimental osteosarcoma models have been widely used to set up new therapeutic protocols and to understand the interactions between osteosarcomas and their environment [10], [11], [12]. In this study, pre-clinical experiments were set up using a human osteosarcoma model induced in athymic nude mice to determine whether tumor growth may be modified by fat grafts. Because we observed that tumor growth was promoted by fat injection, we investigated the role of mesenchymal stem/stromal cells (MSCs), as fat tissue is a well-known source of MSC-like cells whose implication in cancer is controversial.

## Materials and Methods

### Ethics statement

- A surplus of human adipose tissue was used to isolate human mesenchymal stem cells and for injection in mice. This fat surplus was obtained from a female patient in the course of an aesthetic abdominal liposuction. The oral consent of the patient was obtained for the use of fat sample for research. A written consent was not necessary because it was anonymized unlinked research in accordance with French law (Art. L. 1245-2 du code de la santé publique, Loi n° 2004-800 du 6 août 2004 Journal Officiel du 7 août 2004).

- All research involving animals were conducted following the guidelines (named “Charte nationale portant sur l'éthique de l'expérimentation animale”) of the French ethical committee (named “Comité national de réflexion éthique sur l'expérimentation animale”) and have been approved by the committee named CEEA.PdL.06.

### Coleman's procedure

Coleman's procedure was performed over one female patient under general anesthesia [1]. The fat donor site was the abdominal subcutaneous tissues. The fat was taken using a cannula connected to a 10 ml Luer-Lock syringe. A steel stopper device helped to maintain the vacuum in the syringe during the aspiration phase. The sampling syringes were sealed and centrifuged at 3000 rpm for 3 min. After centrifugation the sample was separated into 3 layers: the upper yellow layer composed of oil from destruction of fat fragment, the middle layer composed of the adipose tissue graft and the bottom one composed of blood. The top and bottom layers were discarded and fatty tissue is injected through 1-mm incisions in subcutaneous and muscle tissue to obtain a filling effect.

### Cell culture

#### SaOS2 cell line

The human osteosarcoma cell line SaOS2 was initially derived from an 11-year old Caucasian girl [13]. The cells were cultured in Dulbecco's modified Eagle's medium (DMEM; Biowhittaker, Verviers, Belgium) with 1% antibiotic mixture (Penicillin 100 U/ml and Streptomycin 100 mg/l; Invitrogen, Cergy-Pontoise, France) supplemented with 10% fetal bovine serum (FBS, Dominique Dutscher, Brumath, France), at 37°C in a humidified atmosphere (5% CO_2_/95% air). The cells were harvested at confluence with trypsin (0.5 g/l)/EDTA (0.2 g/l) (Cambrex Bio Sciences, Verviers, Belgium).

#### POS-1 cells

The murine osteosarcoma cell line POS-1, derived from osteosarcoma which spontaneously developed in C3H/HeN mice was kindly provided from Kanagawa Cancer Center (Kanagawa, Japan [14]). The cells were cultured in RPMI 1640 medium (Biowhittaker, Verviers, Belgium) supplemented with 10% FBS, at 37°C in a humidified atmosphere (5% CO_2_/95% air). The cells were harvested at confluence with trypsin (0.5 g/l)/EDTA (0.2 g/l).

#### C3H10T1/2 MSCs

These cells were originally derived from C3H/HeN mouse embryos and were purchased from the American Type Culture Collection (CCL-226, Manassas, VA, USA). They represent a model of murine MSCs, able to differentiate into adipocytes, chondrocytes and osteoblasts [15]. They were cultured in alpha-MEM (Invitrogen, Cergy-Pontoise, France) with 10% FBS.

#### Co-culture

Thirty thousands osteosarcoma POS-1 cells were seeded in 24 well-plates with RPMI 1640 medium supplemented with 10% FBS. Murine C3H10T1/2 MSCs (15 000) were seeded in BD Falcon Cell Culture Insert (pore size 3 microns, BD Biosciences, Erembodegem, Belgium) with alpha-MEM supplemented with 10% FBS. One day after cell seeding, the murine MSCs were incubated with POS-1 cells with RPMI 1640 medium supplemented with 5% FBS. A trypan blue cell counting was then performed on POS-1 cells 24 and 72 hours later.

### Isolation and characterization of human MSCs from adipose tissue

Human fat sample was obtained from one female patient in the course of an aesthetic abdominal liposuction with her informed consent for the use for anonymized unlinked research. The donor had no significant medical history. Fat was removed using the Coleman's procedure and 200 µl of centrifuged fat were plated in 25 cm^2^ flask in 5 ml of alpha-MEM supplemented with 0.5 ml of FBS and 0.05 ml of antibiotics (Penicillin 100 U/ml and Streptomycin 100 mg/l; Invitrogen, Cergy-Pontoise, France). To remove non adherent cells, the cells were washed after 24 hours three times and then supplemented with fresh medium. For phenotypic characterization, surface antigen markers were analyzed by flow cytometry on MSCs. Cells were washed twice with phosphate-buffered solution (PBS) containing 0.4% bovine serum albumin (BSA) before staining with the following antibodies: FITC-conjugated anti-CD90 (Biosciences, Erembodegem, Belgium), PE-conjugated anti-CD105, FITC-conjugated anti-CD34 and PE-conjugated anti-CD45 (Biolegend, San Diego, CA, USA).

#### Differentiation potential assessment

After expansion, the cells were seeded into chamber slides (LabTek, Nunc) and stimulated by osteogenic, adipogenic and vascular smooth muscle inducing media. Osteogenic medium consisted of alpha-MEM medium complemented by 10% FBS, 50 µg/ml freshly prepared ascorbic acid (Sigma, Saint-Quentin, Fallavier, France), 10 mM sodium-beta-glycerophosphate (Sigma) and 300 ng/ml recombinant human BMP4 (RnD systems, Lille, France). The cells were cultured three weeks and then mineralization was assessed by using alizarin red and von Kossa staining [16]. After extensive washing, cells were observed under light microscopy. Adipogenic medium consisted of 1×10-6 M dexamethasone and 0.5 mM isobutylmethylxanthin (IBMX, Sigma). The vascular smooth muscle fate was obtained by adding 20 ng/ml TGFβ1. Nile Red-Oil (Sigma) was used to stained lipid droplets. Monoclonal antibodies recognizing SM-α actin (clone 1A4, Sigma), SM-α actinin (clone 1E12, Developmental Studies Hybridoma Bank, university of Iowa), SM22α (clone 10H12, Novocastra) and SM myosin heavy chain (clone hsmv, Sigma) were used.

### Histological analyses

Surgical specimens were fixed in 10% buffered formaldehyde and after embedding in paraffin, 5 µm-thick sections were stained with haematoxylin–eosin–safran solution. For presence of neutral lipids, osteosarcoma specimens were frozen and sliced into 5 µm-thick sections. Sections were fixed with 10% paraformaldehyde, then rinsed in distilled water, washed in 60% isopropanol and finally stained with Oil Red O solution (3 g/l).

### *In vivo* experiments

#### Human osteosarcoma model in nude mice

Four-week-old female athymic mice (NMRI nu/nu; Elevages Janvier, Le Genest St Isle, France) were housed under pathogen-free conditions at the Experimental Therapy Unit (Faculty of Medicine, Nantes, France) following the guidelines of the French ethical committee (CEEA.PdL.06). The mice were anesthetized by inhalation of an isoflurane-air mix (2%, 1 l/min) before any surgical manipulation. Human osteosarcoma SaOS2 cells were injected in the tibial anterior muscle (8×10^6^ cells in 50 µL PBS) of athymic mice to induce a primary tumor. Then fragments (0.02 g) of this primary tumor were transplanted next to the tibia of mice of the same athymic strain to induce osteosarcoma development [11]. Eight days after fragment transplantation, tumors were detected. Then they were either injected with 100 µl of fat harvested from one female patient following the Coleman's procedure or injured with empty cannula. The tumor volume was calculated by the measure of 2 perpendicular diameters with a vernier caliper and calculated with the formula (l^2^×L)/2 where l and L represent respectively the smallest and the largest diameter.

#### Mouse osteosarcoma model in C3H/HeN mice

Four-week-old male C3H/HeN mice (Elevages Janvier) were housed under pathogen-free conditions at the Experimental Therapy Unit as described above. Subcutaneous injections of POS-1 cells (2×10^6^) were performed in the hind footpad of the mice. In some experiments, POS-1 cells were co-injected with C3H10T1/2 MSCs at the ratio 2/1. The tumor volume was calculated as described above.

## Results

### Case report

**Figure 1:**
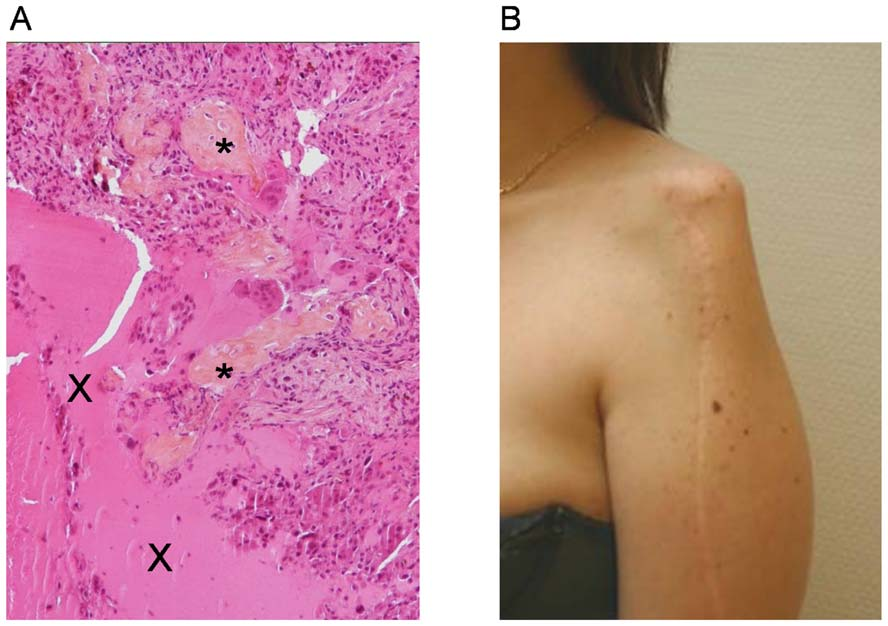
Telangiectasic osteosarcoma and post surgical defect. (**A**) Photomicrograph of the initial telangiectasic osteosarcoma showing proliferation of neoplastic cells, focal osteoïd formation (*) and telangiectasic space containing blood (X) (optical microscopy, HES staining, ×100). (**B**) Post-surgical defect of the left arm in January 2005, 2 months before the first lipofilling procedure.

A 17 year-old girl was admitted in our institution in August 1994 with a metaphyso-epiphyseal bone tumor of the proximal humerus. An incisional biopsy made the diagnosis of telangiectasic osteosarcoma (Fig. 1A), in conformity with clinical and radiological features. The patient received pre operative chemotherapy in respect to french protocol at this time (4 cures of Holoxan/cis platinium). Then a limb-salvage procedure consisting of an extra articular resection of proximal humerus with gleno-humeral articulation was performed. The reconstruction consisted of an arthrodesis with a vascularised scapular crest bone graft. The histopathologic examination of the specimen concluded of clear wide margins and no viable cells have been found out (good response, Huvos grade 4). The patient received two more cures of the same chemotherapeutic protocol. She has been continuously follow-up every 6 months for 5 years then every year [Magnetic Resonance Imaging (MRI) and chest X ray] until now.

In 2005, because of the unsightly appearance of her left arm due to resection of soft tissue and mainly deltoid muscle (Fig. 1B), she was referred to a reconstructive surgeon of our institution and received three autologous fat grafts harvested from her abdominal wall and injected circularly around the bone graft in soft tissues (March 2005, June 2005 and January 2006). The patient reported a good cosmetic result with a lasting increased volume of the arm.

**Figure 2:**
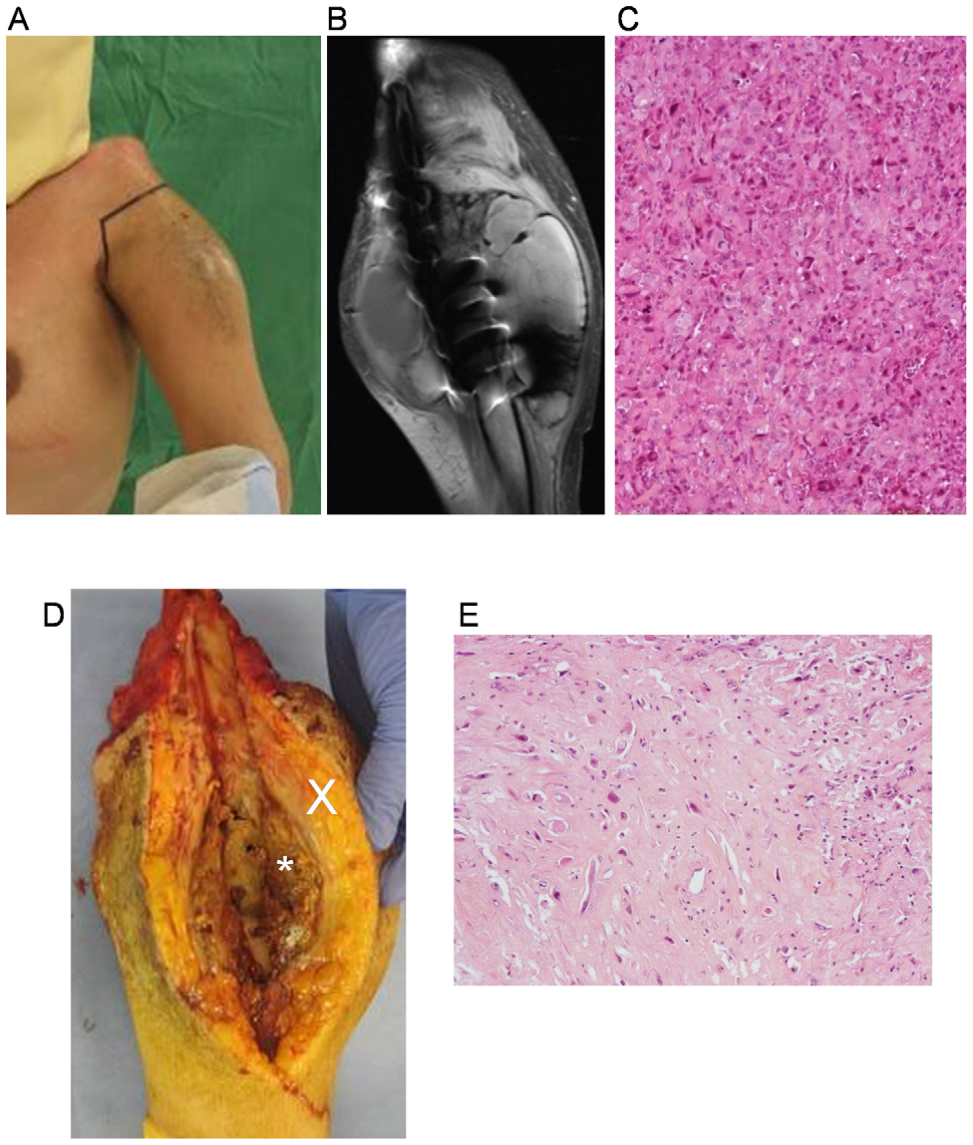
Osteosarcoma recurrence at the lipofilling site. (**A**) Inflammatory and swelling of the lipofilling site at the left arm after 4 cures of chemotherapy. (**B**) T2 weighted MRI shows extensive circular soft tissue tumor of the left arm, measuring 12 cm by 10 cm. (**C**) Photomicrograph of the biopsy specimen at the recurrent tumor site showing high grade malignant cell proliferation and thin osteoïd formation (optical microscopy, HES staining, ×100). (**D**) Photograph of the open resected tumor with tumor mass (*) around the left humerus diaphisis and peripheral fat engraftment (X) (**E**) Histology of resected tumor specimen (optical microscopy, HES staining, ×100).

In August 2007, she consulted her oncologist because of pain, inflammation and swelling at the lipofilling site (Fig. 2A). The Magnetic Resonance Imaging (MRI) showed an extensive soft tissue tumor (Fig. 2B). Histological analysis of the biopsy revealed an osteosarcoma with neoplastic cells and thin osteoïd formation (Fig. 2C). No lipid droplets have been observed after Oil Red O staining of the biopsy. No distant relapses have been found out. The patient received again 4 cures of chemotherapy (API-AI French protocol) and a trans scapula amputation was performed (Fig. 2D). Tumor cells still responded well to chemotherapy (Huvos grade 3) and no post operative chemotherapy was proposed because of severe renal deficiency after pre operative chemotherapy. Despite the high rate of necrosis, the pathologist was able to see wide area of haemorrhage compatible with telangiectasic subtype (Fig. 2E). One year after the surgery the patient does not present any sign of local or distant relapse.

### Osteosarcoma growth is increased by fat injection

**Figure 3:**
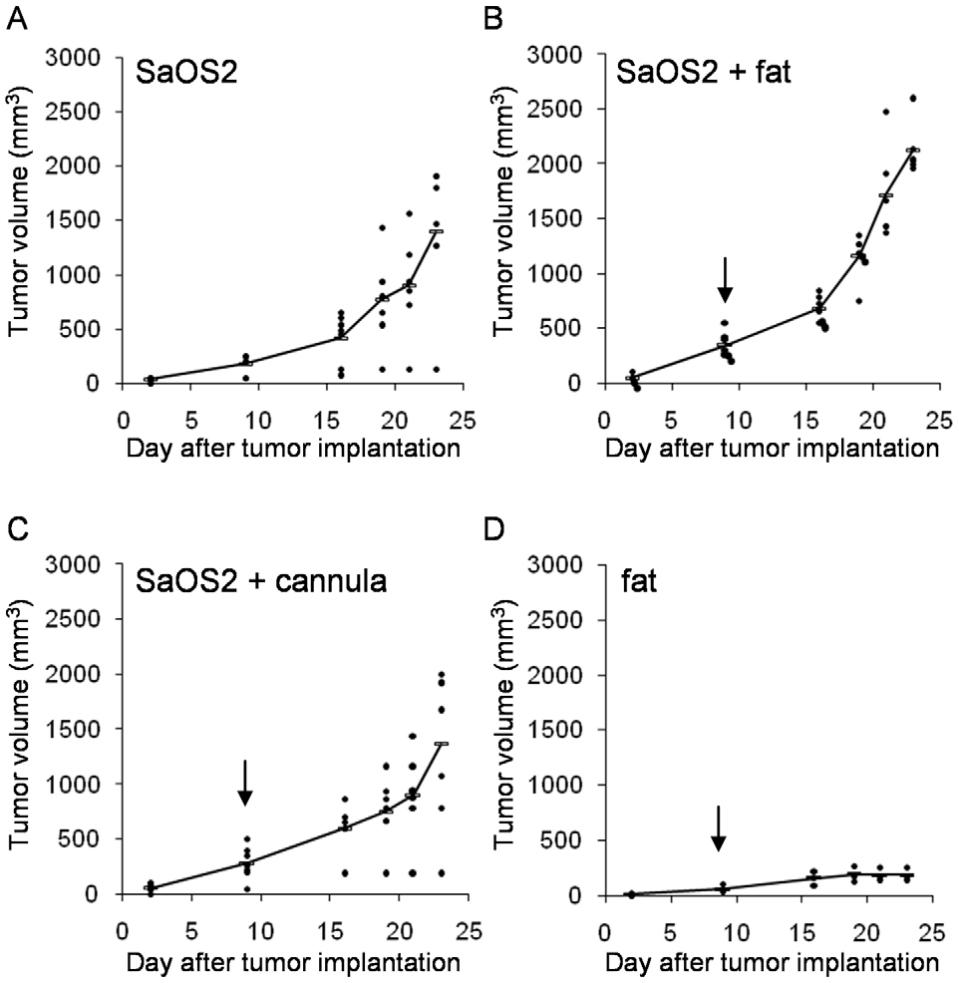
Fat injections into osteosarcoma induced in nude mice. (**A**) Six mice were considered as control tumors (SaOS2). (**B**) A group of 6 mice was injected at the tumor site with human fat harvested by the Coleman's technique (SaOS2 + fat). (**C**) A control group of 6 mice was injured with empty cannula at the tumor site. (**D**) Another group of 3 mice received the fat alone. Arrows indicate day 8 after transplantation when fat injections or cannula injuries were performed. Statistical analyses were calculated with the Kruskal-Wallis test (nonparametric ANOVA) using GraphPad InStat v3.02 software. The p value was less than 0.05 for SaOS2 + fat versus SaOS2 alone or versus SaOS2 + cannula at day 21, but was not significant for SaOS2 alone versus SaOS2 + cannula.

As a result of this surprising clinical case, we wondered whether fat graft was the cause of the local late osteosarcoma recurrence. In a first attempt to test this hypothesis, we tested whether a fat sample which is used for an autologous fat graft can modify the growth of human osteosarcoma induced in immunodeficient mice. First, athymic nude mice were injected with human SaOS2 osteosarcoma cells in muscle leading to tumor development in less than 50% of mice. To obtain a greater bone tumor incidence, fragments of a primary tumor induced by SaOS2 cell injection were transplanted next to the tibia of recipient mice of the same strain. Twenty one mice were transplanted with human osteosarcoma fragments of the same weight, then were divided into 3 groups: the control SaOS2 group just received tumor fragment at day 0; the SaOS2 + fat group was injected with human fat into the tumor site at day 8 after transplantation when a progressive tumor was detected (size >100 mm^3^); the SaOS2 + cannula group was transplanted with tumor fragment at day 0, then at day 8 the tumors were injured with an empty cannula to reproduce potential inflammatory process; and a group of three mice was injected only with human fat in the tibial muscle. Results of tumor progression are shown in figure 3. In the control SaOS2 group, osteosarcoma developed rapidly reaching a mean volume of 1393 mm^3^ 23 days after tumor implantation (Fig. 3A). Tumor growth was greatly and significantly increased at day 21 after tumor induction in the group that received fat injections (Fig. 3B). As the fat injection was realized with a cannula which may have induced inflammation, a group of injured osteosarcoma was studied in the same conditions. In this case, the inflammation caused by cannula injury did not change osteosarcoma growth as compared to the control SaOS2 group with uninjured osteosarcoma (Fig. 3C). The tumor size increase observed in the SaOS2 + fat group was not due to the fat mass itself as only reduced and stable volumes (less than 200 mm^3^) were observed with fat injections alone into naive control mice (Fig. 3D). Moreover, we could observe that animals which received fat grafts in osteosarcoma tumors were more homogeneous in their tumor progression as compared with control groups (SaOS2 and SaOS2 + cannula).

**Figure 4:**
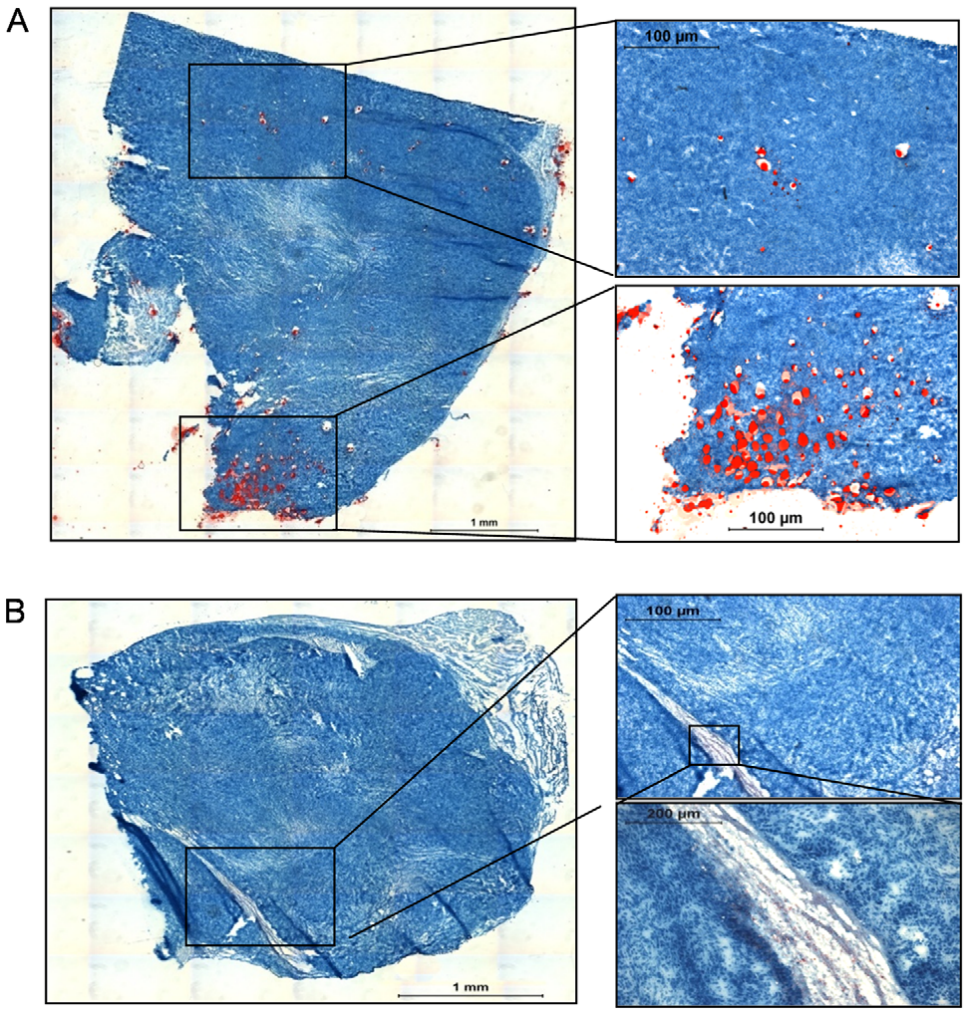
Identification of lipid droplets in osteosarma specimens. (**A**) Photomicrograph of a tumor specimen from the SaOS2 + fat group showing large red lipid droplets. (**B**) Photomicrograph of a tumor specimen from the SaOS2 + cannula group showing small red lipid droplets (optical microscopy, Oil Red O staining).

Histological analyses revealed few but large adipocytes between the neoplastic cells for tumor specimens of the SaOS2 + fat group (Fig. 4A), whereas only small adipocytes were identified within fibrotic areas and not between neoplastic cells within tumor specimens of the SaOS2 + cannula group (Fig. 4B) or control SaOS2 group (data not shown). The proportion of these large adipocytes seems too small to explain the tumor size increase which was observed in the SaOS2 + fat group. Then we may hypothesize that fat injections have stimulated osteosarcoma cell proliferation.

### MSC-like cells are present in the injected fatty tissue

**Figure 5:**
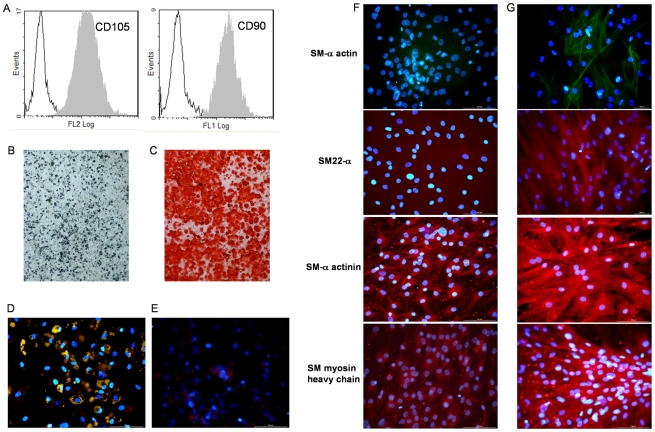
Characterisation of human fat derived MSC-like cells. (**A**) Flow cytometry analysis showed CD105 and CD90 positive cells after the third passage in culture. In addition, multipotential capacities of MSC-like cells were tested by inducing cells to differentiate into osteoblastic cells capable to mineralize, adipocytes and vascular smooth muscle (VSM) cells. (**B**) Mineralized nodules were identified as black spots using von Kossa silver staining or (**C**) as red spots using alizarin red staining after incubation of fat derived MSC-like cells in osteogenic differentiation medium. Adipocytes containing small Nil Red-positive lipid droplets were easily observed upon adipogenic conditions (**D**) whereas non induced cells were not labeled (**E**). In contrast to non induced cells (**F**), those cultured upon VSM conditions (**G**) generated elongated cells positive for several VSM markers such as SM-α actin, SM22α, SM-α actinin and SM myosin heavy chain.

One hypothesis for the stimulation of osteosarcoma growth by fat injection may lie to the presence of MCSs in the injected fatty tissue. An aliquot of the fat tissue which was injected into SaOS2-bearing mice was tested in culture for the presence of adherent cells. Such cells were obtained and analyzed by flow cytometry. At passage 3, these cells were positive for the MSC markers CD105 and CD90 (Fig. 5A) and simultaneously negative for the hematopoietic markers CD45 and CD34 (data not shown). Moreover, these cells differentiated into the osteoblastic and adipocytic cells as well as muscle cells from vascular smooth muscle lineage as shown in figure 5. Indeed, according to the type of induction the presence of mineralized nodules was identified by either von Kossa or alizarin red staining and adipocytes by their content in lipid droplets positives labelled by Nil Red-Oil. In addition, MSC-like cells were able to give rise vascular smooth muscle (VSM) cells since expression of cytoskeletal molecules specific of VSM lineage such as SM22α, SM myosin heavy chain, SM-α actin and SM-α actinin increased dramatically after VSM induction. Therefore, adherent cells-derived from fat had characteristics similar to what it is largely described for bone marrow MSC [17].

After passage 8, these MSC-like cells were injected in three mice and the three intramuscular injections of two million cells per mouse did not induce tumor development after 60 days. These fat-derived cells maintained long-term self-renewal (>20 passages), but they have lost the CD105 expression as early as passage 6 showing rapid phenotypic change in culture.

### Injections of MSCs with osteosarcoma cells increase tumor progression

**Figure 6:**
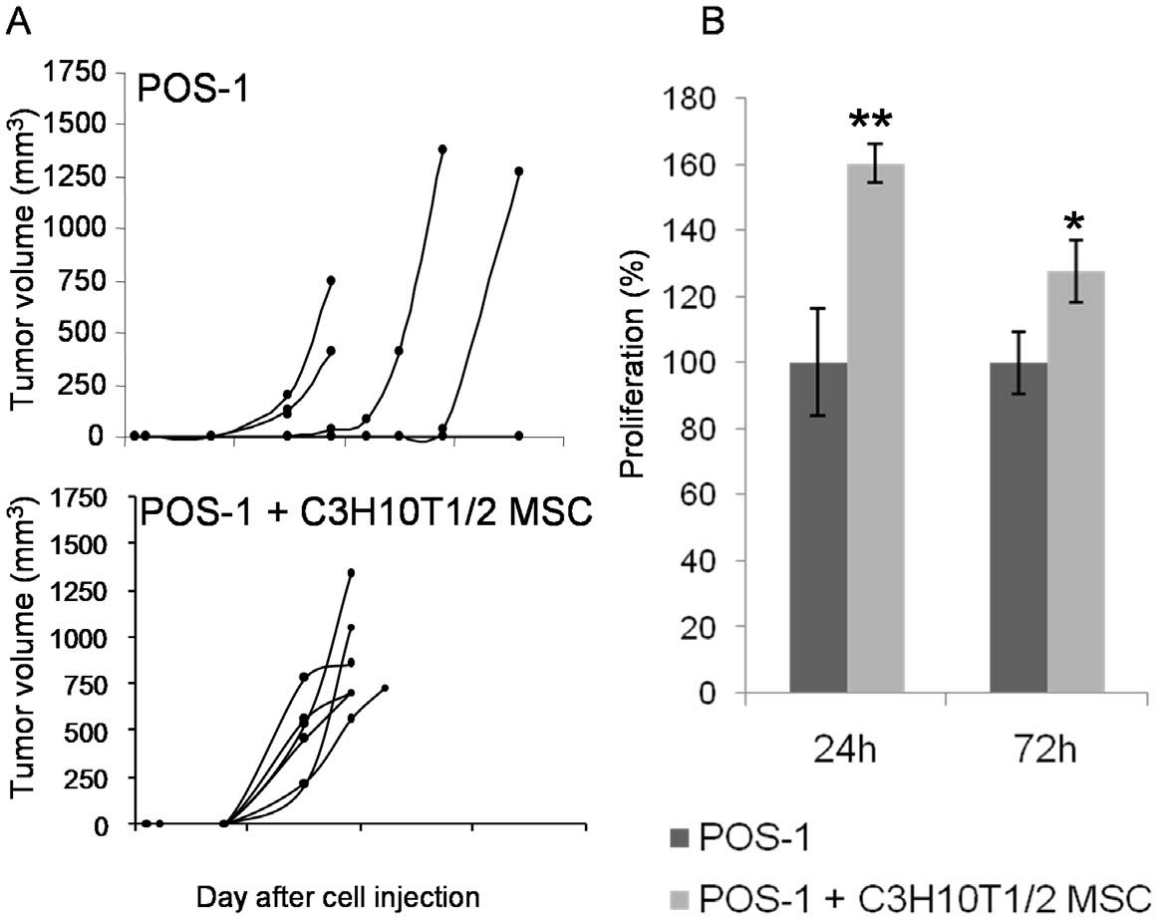
Mouse MSCs interact with osteosarcoma cells. (**A**) Evolutions of the tumor volume induced into the footpad of C3H/HeN mice. Mice of the control group (POS-1) received osteosarcoma POS-1 cells alone, while POS-1 cells were associated with C3H10T1/2 MSCs at a ratio of 2∶1 in the POS-1 + C3H101/2 MSC group. (**B**) POS-1 osteosarcoma cells and C3H/10T1/2 MSCs were co-cultured without cell-cell contact. The POS-1 cell proliferation was analyzed by trypan blue cell counting after 24 and 72 hours of co-culture. Results are presented as proliferation percentages relatively to the total number of POS-1 cells cultured alone. Error bars represent standard deviations and asterisks indicate significant differences between means (* for p<0.05 and ** for p<0.01).

To determine whether MSCs can interact with osteosarcoma cells and may modulate the early tumor development, we compared the tumor progression induced in mice by injection of osteosarcoma cells alone or with MSCs. For this experiment, we tested mouse osteosarcoma POS-1 cells rather than human SaOS2 cells because their injection in mouse footpad led to a greater tumor indidence (80% versus 50%). The POS-1 cells and C3H10T1/2 MSCs have been derived from the C3H/HeN mouse strain, allowing us to perform this experiment in a syngenic immunocompetent model. Eighteen C3H/HeN mice were divided into 3 groups: one control group was injected with 2×10^6^ osteosarcoma POS-1 cells (POS-1 group); a second control group received 10^6^ C3H10T1/2 cells (C3H10T1/2 MSC group); and mice in the test group were co-injected with POS-1 and C3H10T1/2 cells at the above mentioned concentrations (POS-1 + C3H10T1/2 MSC). Results of individual tumor progression showed a disparity in the time course of tumor onset in the POS-1 group (Fig. 6A): 5 animals out of 6 developing a tumor at different time (from day 15 to 32). On the contrary, all the animals in the POS-1 + C3H10T1/2 MSC group developed a tumor early after cell injection (around day 10) with the same time of onset (Fig. 6A); while MSCs alone did not induce any tumor development (data not shown). The tumor development appeared earlier and more homogenously in the POS-1 + C3H10T1/2 MSC group than in the POS-1 group. The median tumor volume was three times higher in the POS-1 + C3H10T1/2 MSC group than the one in the POS-1 group at day 19 (775 versus 218.5 mm^3^) and the difference was very significant (p = 0.0076 with Mann-Whitney test using GraphPad InStat v3.02 software). Similar results were obtained using a co-injection of rat MSC-like cells and rat osteosarcoma cells in the footpad of nude mice: earlier onset and faster growth of tumors as compared to osteosarcoma cells injected alone (Figure S1A).

We wondered whether MSCs may change the proliferation rate of osteosarcoma cells. Then C3H10T1/2 MSCs and POS-1 cells were co-cultured *in vitro* at a 1∶2 ratio without cell contact. The number of blue stained POS-1 cells did not change when comparing co-cultured and cultured alone POS-1 cells; whereas the number of POS-1 live cells was significantly increased when co-cultured with C3H10T1/2 MSCs as compared to POS-1 cultured alone (Fig. 6B). Similarly the proliferation of rat osteosarcoma cells (OSRGA) was increased when co-cultured with rat MSC-like cells under same experimental conditions as described above (Figure S1B). Therefore MSC-like cells secreted one or several soluble factors which have promoted osteosarcoma cell proliferation *in vitro*.

## Discussion

A patient in complete remission of an humerus telangiectasic osteosarcoma since ten years consulted for a cosmetic correction by lipofilling. Three injections of autologous fat grafts harvested by the Coleman's technique were realized and few months after the last one, a local recurrence of osteosarcoma occurred 13 years after the initial treatment. Late local recurrence of an osteosarcoma is a very unexpected event after complete remission and 10 years of follow-up. To our knowledge this is one of the longest delay reported between remission and local recurrence, another case being reported by Bacci *et al*. [18]. The patient reported here did not present any predictive factor of local recurrence. It was a telangiectasic osteosarcoma subtype and wide margins were obtained after extra articular monobloc resection, both considered as good predictive factors of disease free survival [8], [18]. Even a bad response to chemotherapy is not considered as a bad prognostic factor for late relapse (after 4 years) instead of earlier relapse [19], [20]. Thus, this local recurrence of telangiectasic osteosarcoma at the exact area of autologous fat grafts performed 18 months before raised the question of their possible relationship. Despite the widespread clinical use of Coleman's lipofilling technique, very little is known about its consequence on tissue environment after tumor resection. The only published data concerned conservative treatments in breast cancer patients in which this technique represents an advance, but in these cases the correlation between fat transfer and recurrent breast cancer is difficult to analyze [2], [21], [22], [23], [24]. Motrescu and Rio have described a vicious cycle between adipocytes and cancer cells: the last ones induce secretion by adipose tissue of Matrix Metalloproteinase 11 which in turn favors cancer cell survival and tumor progression by changing the tumor surrounding environment [25]. To investigate the possible relationship between fat graft and osteosarcoma growth, an experimental model reproducing the clinical case of human fat injection in osteosarcoma was set up using human SaOS2 osteosarcoma cells in athymic mice. The human fat tissue was harvested from a patient and injected following the Coleman's procedure. Results showed that osteosarcoma growth was significantly increased by fat injection whereas fat alone produced only small and stable volumes at injection sites in naive mice. Several hypotheses could explain these observations. On one hand, fat may promote angiogenesis thereby interfering with tumor growth. Indeed, Vascular Endothelial Growth Factor (VEGF) which is expressed in the interstitial connective tissue of fat grafts favours graft survival [26], but can stimulate growth, invasion and metastasis of solid tumors [27], [28]. In another hand, adipocytes highly express Adiponectin (also designed as adipose most abundant gene transcript) which was shown to stimulate Receptor Activator of NF-κB Ligand (RANKL or TNFSF11) and inhibit Osteoprotegerin expression in osteoblasts [29]. Therefore fat may regulate two bone resorption mediators which are highly implicated in the development of bone tumors [30]. Indeed blocking RANKL by soluble RANK [31], [32], [33] or by the decoy receptor Osteoprotegerin [11], [34], [35], [36], [37] was proven to be effective in several *in vivo* models of bone tumors and leads to the clinical development of a fully-humanized monoclonal antibody directed against RANKL (Denosumab) [38].

In addition, the role of MSC-like cells which are contained in fatty tissue must be considered [39]. Our study showed that the fat harvested following Coleman's procedure contains progenitor cells which share numerous characteristics with MSCs. They were positive for the phenotypic markers CD90 and CD105 and simultaneously negative for the hematopoietic markers CD45 and CD34. They showed ability to survive in long term culture without producing tumor *in vivo* and to differentiate *in vitro* into the osteoblast lineage. Adipose-derived stem/stromal cells (designed ADSCs or ASCs) are proposed as cellular agent to enhance angiogenesis after ischemic injury [40], [41] or to promote survival of fat graft for breast augmentation or reconstruction [42], but additionally they could promote migration and invasion of breast cancer cells [43]. The effect of bone marrow-derived MSCs has been studied on osteosarcoma development by injection in the caudal vein of nude mice bearing SaOS2 cell-induced osteosarcoma: the addition of MSCs has promoted tumor growth and pulmonary metastasis *in vivo* [44]. In our study, we used a complete syngeneic model with POS-1 osteosarcoma cells and C3H10T1/2 MSCs which have been derived from C3H/HeN mice and were injected into mice of the same strain. The osteosarcoma onset and incidence were greatly enhanced by C3H10T1/2 MSC presence. Djouad F *et al.* have shown an immunosuppressive effect of primary or C3H10T1/2 MSCs on tumor growth: B16 melanoma cells have proliferated into allogeneic C3H/HeN mice only when they have been co-injected with MSCs otherwise they have been rejected [45]. Into a syngeneic tumor model, C3H10T1/2 MSCs have induced an earlier onset of tumors but did not interfere with the tumor growth kinetic [46]. Using a co-culture method without cell contact, we observed that C3H10T1/2 MSCs promoted POS-1 cell proliferation by secreting one or several soluble factors. We noted similar observations like earlier onset of tumors in a xenogeneic model and greater proliferation *in vitro* when rat osteosarcoma cells were combined with rat MSC-like cells. Numerous soluble factors have to be considered as MSC mediators able to modify tumor cell proliferation or the tumor environment. Among them, the insulin growth factor, the fibroblast growth factor, the interleukin Il-6, the chemokine CCL5 (RANTES) and the matrix metalloproteinases MMP-2 and MT1-MMP are few of the mediators that could have a particular role in bone tumor development [47], [48], [49]. Further studies have to be performed to identify the MSC mediators acting on osteosarcoma cells. To address this point, the main difficulty is that the panel of cytokines and growth factors secreted by MSCs is likely to be different *in vitro* and *in vivo* because their expression can be modulated by inflammatory mediators *in vivo* [50].

Controversial results have been reported when MSCs have been injected into mice with induced tumor, resulting either in promotion or inhibition of tumor growth depending on the studies. Antitumoral action of rat MSCs on rat gliomas has been reported by Nakamura *et al.* [51]. In that report, MSCs have been responsible for prolonged survival of glioma-bearing rats. Similarly and somewhat to their own surprise, Khakoo AY *et al.* [52] have obtained also an antitumorigenic effect of human MSCs on Kaposi's sarcomas which have been induced on nude mice. Since their first warning, Djouad F *et al.* have also reported that the immunosuppressive effect of mouse MSCs *in vivo* depends on environmental parameters and that even “a low but relevant amount of MSCs may induce tumor rejection” [46]. Therefore the effect of MSCs within tumors seems unpredictable and dependant of microenvironment signals. By exploring the interaction of MSCs with the tumor environment, new therapeutic options could be provided [53].

In an osteosarcoma experimental model, Naumov *et al.* have shown that switch from non angiogenic to angiogenic phenotype could be a biologic behavior associated to the clinical expression of a quiescent tumor [54]. Assuming that cancer stem cell-like theory in bone sarcoma is correct [55], [56], their presence at the site of the primary tumor after treatment is in agreement with a “supposed” good response chemotherapy and wide monobloc surgery as in our patient. Despite the lack of biological data on the mechanism by which cancer stem cells drive the tumor growth, the modulation of microenvironment induced by the fat graft could interfere with the biological behaviour of this sub-population. The preclinical experimental designs used in the present study mimic surgical procedure for lipofilling enabling to study *in vivo* interaction between fat and osteosarcoma cells, but do not mimic the late and local recurrence of osteosarcoma after a long lasting remission as observed in the clinical case reported here. Further experimental investigations are necessary to understand the mechanism regulating the interactions between the graft, the tissue recipient and quiescent tumor cells. However surgery associating inflammatory process could be directly responsible for the reactivation of dormant tumor cells [57], [58]. This last assumption attributes a minor or non role to adipose tissue but constitutes anyway a warning concerning the fat grafting or any other surgical procedure in a post-neoplastic environment.

In conclusion, because of the unexpected late local recurrence of an osteosarcoma at the exact area of autologous fat grafts and the stimulation of osteosarcoma tumor growth by fat or MSC-like cells in experimental models, clinician must be aware of the possible long term local relapse of tumor after an autologous fat graft which is usually considered as a safe procedure.

## Supporting Information

Figure S1 Rat MSCs interact with osteosarcoma cells. (A) Evolutions of the tumor volume induced into the footpad of nude mice (NMRI nu/nu; Elevages Janvier, Le Genest St Isle, France). The OSRGA cells were derived from a transplantable rat osteosarcoma model originally induced by radiation [11]. Relatively undifferentiated mesenchymal cells (MSC-like cells) were obtained from calvaria of newborn rat (2 days old Sprague-Dawley rat, Charles River, L'Arbresle, France) as previously described. Mice of the control group (OSRGA) received 106 OSRGA cells alone, while OSRGA cells were associated with calvaria-derived MSC-like cells at ratio 2∶1 in the OSRGA + MSC group. A third group received 0.5×106 MSC-like cells alone and have not developed any tumor after 85 days (data not shown). The significance test is not performed as only three mice per group were included in this preliminary experiment. (B) OSRGA cells and calvaria-derived MSC-like cells were co-cultured without cell-cell contact. The OSRGA cell proliferation was analyzed by trypan blue cell counting after 24 and 72 hours. Results are presented as proliferation percentages relatively to the total number of OSRGA cells cultured alone. Error bars represent standard deviations and asterisks indicate significant differences between means (p<0.01).
(0.50 MB TIF)

## References

[1] Coleman SR (1997). Facial recontouring with lipostructure.. Clin Plast Surg.

[2] Coleman SR, Saboeiro AP (2007). Fat grafting to the breast revisited: safety and efficacy.. Plast Reconstr Surg.

[3] Spear SL (2008). Fat for breast: where are we?. Plast Reconstr Surg.

[4] Missana MC, Laurent I, Barreau L, Balleyguier C (2007). Autologous fat transfer in reconstructive breast surgery: indications, technique and results.. Eur J Surg Oncol.

[5] Salgarello M, Visconti G, Farallo E (2009). Autologous Fat Graft in Radiated Tissue Prior to Alloplastic Reconstruction of the Breast: Report of Two Cases.. Aesthetic Plast Surg.

[6] Ferrari S, Bacci G, Picci P, Mercuri M, Briccoli A (1997). Long-term follow-up and post-relapse survival in patients with non-metastatic osteosarcoma of the extremity treated with neoadjuvant chemotherapy.. Ann Oncol.

[7] Meyers PA, Heller G, Healey J, Huvos A, Lane J (1992). Chemotherapy for nonmetastatic osteogenic sarcoma: the Memorial Sloan-Kettering experience.. J Clin Oncol.

[8] Bielack SS, Kempf-Bielack B, Delling G, Exner GU, Flege S (2002). Prognostic factors in high-grade osteosarcoma of the extremities or trunk: an analysis of 1,702 patients treated on neoadjuvant cooperative osteosarcoma study group protocols.. J Clin Oncol.

[9] Koksal Y, Akyuz C, Varan A, Atilla B, Gedikoglu G (2008). Late recurrence in primary region of parosteal osteosarcoma: a case report.. Pediatr Hematol Oncol.

[10] Lamoureux F, Picarda G, Garrigue-Antar L, Baud'huin M, Trichet V (2009). Glycosaminoglycans as potential regulators of osteoprotegerin therapeutic activity in osteosarcoma.. Cancer Res.

[11] Lamoureux F, Richard P, Wittrant Y, Battaglia S, Pilet P (2007). Therapeutic relevance of osteoprotegerin gene therapy in osteosarcoma: blockade of the vicious cycle between tumor cell proliferation and bone resorption.. Cancer Res.

[12] Ory B, Heymann MF, Kamijo A, Gouin F, Heymann D (2005). Zoledronic acid suppresses lung metastases and prolongs overall survival of osteosarcoma-bearing mice.. Cancer.

[13] Fogh J, Fogh JM, Orfeo T (1977). One hundred and twenty-seven cultured human tumor cell lines producing tumors in nude mice.. J Natl Cancer Inst.

[14] Uesugi M, Koshino T, Mitsugi N, Hiruma T (2000). Predictive value of serum immunosuppressive acidic protein for lung metastasis after amputation of murine osteosarcoma of the lower limb.. Cancer Lett.

[15] Pinney DF, Emerson CP (1989). 10T1/2 cells: an in vitro model for molecular genetic analysis of mesodermal determination and differentiation.. Environ Health Perspect.

[16] Bills CE, Eisenberg H, Pallante SL (1971). Complexes of organic acids with calcium phosphate: the von Kossa stain as a clue to the composition of bone mineral.. Johns Hopkins Med J.

[17] Dennis JE, Charbord P (2002). Origin and differentiation of human and murine stroma.. Stem Cells.

[18] Bacci G, Ferrari S, Bertoni F, Ruggieri P, Picci P (2000). Long-term outcome for patients with nonmetastatic osteosarcoma of the extremity treated at the istituto ortopedico rizzoli according to the istituto ortopedico rizzoli/osteosarcoma-2 protocol: an updated report.. J Clin Oncol.

[19] Ferrari S, Briccoli A, Mercuri M, Bertoni F, Cesari M (2006). Late relapse in osteosarcoma.. J Pediatr Hematol Oncol.

[20] Kim MS, Cho WH, Song WS, Lee SY, Jeon DG (2007). time dependency of prognostic factors in patients with stage II osteosarcomas.. Clin Orthop Relat Res.

[21] Chan CW, McCulley SJ, Macmillan RD (2008). Autologous fat transfer–a review of the literature with a focus on breast cancer surgery.. J Plast Reconstr Aesthet Surg.

[22] Delay E, Gosset J, Toussoun G, Delaporte T, Delbaere M (2008). [Efficacy of lipomodelling for the management of sequelae of breast cancer conservative treatment].. Ann Chir Plast Esthet.

[23] Gosset J, Flageul G, Toussoun G, Guerin N, Tourasse C (2008). [Lipomodelling for correction of breast conservative treatment sequelae. Medicolegal aspects. Expert opinion on five problematic clinical cases].. Ann Chir Plast Esthet.

[24] Mojallal A, Saint-Cyr M, Garrido I (2009). Autologous fat transfer: controversies and current indications for breast surgery.. J Plast Reconstr Aesthet Surg.

[25] Motrescu ER, Rio MC (2008). Cancer cells, adipocytes and matrix metalloproteinase 11: a vicious tumor progression cycle.. Biol Chem.

[26] Nishimura T, Hashimoto H, Nakanishi I, Furukawa M (2000). Microvascular angiogenesis and apoptosis in the survival of free fat grafts.. Laryngoscope.

[27] Kaya M, Wada T, Akatsuka T, Kawaguchi S, Nagoya S (2000). Vascular endothelial growth factor expression in untreated osteosarcoma is predictive of pulmonary metastasis and poor prognosis.. Clin Cancer Res.

[28] Vona-Davis L, Rose DP (2009). Angiogenesis, adipokines and breast cancer.. Cytokine Growth Factor Rev.

[29] Luo XH, Guo LJ, Xie H, Yuan LQ, Wu XP (2006). Adiponectin stimulates RANKL and inhibits OPG expression in human osteoblasts through the MAPK signaling pathway.. J Bone Miner Res.

[30] Kingsley LA, Fournier PG, Chirgwin JM, Guise TA (2007). Molecular biology of bone metastasis.. Mol Cancer Ther.

[31] Lamoureux F, Picarda G, Rousseau J, Gourden C, Battaglia S (2008). Therapeutic efficacy of soluble receptor activator of nuclear factor-kappa B-Fc delivered by nonviral gene transfer in a mouse model of osteolytic osteosarcoma.. Mol Cancer Ther.

[32] Pearse RN, Sordillo EM, Yaccoby S, Wong BR, Liau DF (2001). Multiple myeloma disrupts the TRANCE/osteoprotegerin cytokine axis to trigger bone destruction and promote tumor progression.. Proc Natl Acad Sci U S A.

[33] Zhang J, Dai J, Yao Z, Lu Y, Dougall W (2003). Soluble receptor activator of nuclear factor kappaB Fc diminishes prostate cancer progression in bone.. Cancer Res.

[34] Croucher PI, Shipman CM, Lippitt J, Perry M, Asosingh K (2001). Osteoprotegerin inhibits the development of osteolytic bone disease in multiple myeloma.. Blood.

[35] Heath DJ, Vanderkerken K, Cheng X, Gallagher O, Prideaux M (2007). An osteoprotegerin-like peptidomimetic inhibits osteoclastic bone resorption and osteolytic bone disease in myeloma.. Cancer Res.

[36] Jones DH, Nakashima T, Sanchez OH, Kozieradzki I, Komarova SV (2006). Regulation of cancer cell migration and bone metastasis by RANKL.. Nature.

[37] Morony S, Capparelli C, Sarosi I, Lacey DL, Dunstan CR (2001). Osteoprotegerin inhibits osteolysis and decreases skeletal tumor burden in syngeneic and nude mouse models of experimental bone metastasis.. Cancer Res.

[38] Schwarz EM, Ritchlin CT (2007). Clinical development of anti-RANKL therapy.. Arthritis Res Ther.

[39] Gimble JM, Nuttall ME (2004). Bone and fat: old questions, new insights.. Endocrine.

[40] Carriere A, Ebrahimian TG, Dehez S, Auge N, Joffre C (2009). Preconditioning by mitochondrial reactive oxygen species improves the proangiogenic potential of adipose-derived cells-based therapy.. Arterioscler Thromb Vasc Biol.

[41] Rehman J, Traktuev D, Li J, Merfeld-Clauss S, Temm-Grove CJ (2004). Secretion of angiogenic and antiapoptotic factors by human adipose stromal cells.. Circulation.

[42] Yoshimura K, Sato K, Aoi N, Kurita M, Hirohi T (2008). Cell-assisted lipotransfer for cosmetic breast augmentation: supportive use of adipose-derived stem/stromal cells.. Aesthetic Plast Surg.

[43] Walter M, Liang S, Ghosh S, Hornsby PJ, Li R (2009). Interleukin 6 secreted from adipose stromal cells promotes migration and invasion of breast cancer cells.. Oncogene.

[44] Xu WT, Bian ZY, Fan QM, Li G, Tang TT (2009). Human mesenchymal stem cells (hMSCs) target osteosarcoma and promote its growth and pulmonary metastasis.. Cancer Lett.

[45] Djouad F, Plence P, Bony C, Tropel P, Apparailly F (2003). Immunosuppressive effect of mesenchymal stem cells favors tumor growth in allogeneic animals.. Blood.

[46] Djouad F, Bony C, Apparailly F, Louis-Plence P, Jorgensen C (2006). Earlier onset of syngeneic tumors in the presence of mesenchymal stem cells.. Transplantation.

[47] Damiens C, Grimaud E, Rousselle AV, Charrier C, Fortun Y (2000). Cysteine protease production by human osteosarcoma cells (MG63, SAOS2) and its modulation by soluble factors.. Cytokine.

[48] Georges S, Ruiz Velasco C, Trichet V, Fortun Y, Heymann D (2009). Proteases and bone remodelling.. Cytokine Growth Factor Rev.

[49] Lazennec G, Jorgensen C (2008). Concise review: adult multipotent stromal cells and cancer: risk or benefit?. Stem Cells.

[50] Ponte AL, Marais E, Gallay N, Langonne A, Delorme B (2007). The in vitro migration capacity of human bone marrow mesenchymal stem cells: comparison of chemokine and growth factor chemotactic activities.. Stem Cells.

[51] Nakamura K, Ito Y, Kawano Y, Kurozumi K, Kobune M (2004). Antitumor effect of genetically engineered mesenchymal stem cells in a rat glioma model.. Gene Ther.

[52] Khakoo AY, Pati S, Anderson SA, Reid W, Elshal MF (2006). Human mesenchymal stem cells exert potent antitumorigenic effects in a model of Kaposi's sarcoma.. J Exp Med.

[53] Mishra PJ, Mishra PJ, Glod JW, Banerjee D (2009). Mesenchymal stem cells: flip side of the coin.. Cancer Res.

[54] Naumov GN, Bender E, Zurakowski D, Kang SY, Sampson D (2006). A model of human tumor dormancy: an angiogenic switch from the nonangiogenic phenotype.. J Natl Cancer Inst.

[55] Gibbs CP, Kukekov VG, Reith JD, Tchigrinova O, Suslov ON (2005). Stem-like cells in bone sarcomas: implications for tumorigenesis.. Neoplasia.

[56] Wilson H, Huelsmeyer M, Chun R, Young KM, Friedrichs K (2008). Isolation and characterisation of cancer stem cells from canine osteosarcoma.. Vet J.

[57] Demicheli R, Retsky MW, Hrushesky WJ, Baum M (2007). Tumor dormancy and surgery-driven interruption of dormancy in breast cancer: learning from failures.. Nat Clin Pract Oncol.

[58] Varani J, Lovett EJ, Lundy J (1981). A model of tumor cell dormancy: effects of anesthesia and surgery.. J Surg Oncol.

