# Supplementary figures and images for: Safety Concern between Autologous Fat Graft, Mesenchymal Stem Cell and Osteosarcoma Recurrence
Source: PLoS One. 2010 Jun 8;5(6):e10999. doi: 10.1371/journal.pone.0010999 (PMC2882323; doi:10.1371/journal.pone.0010999)

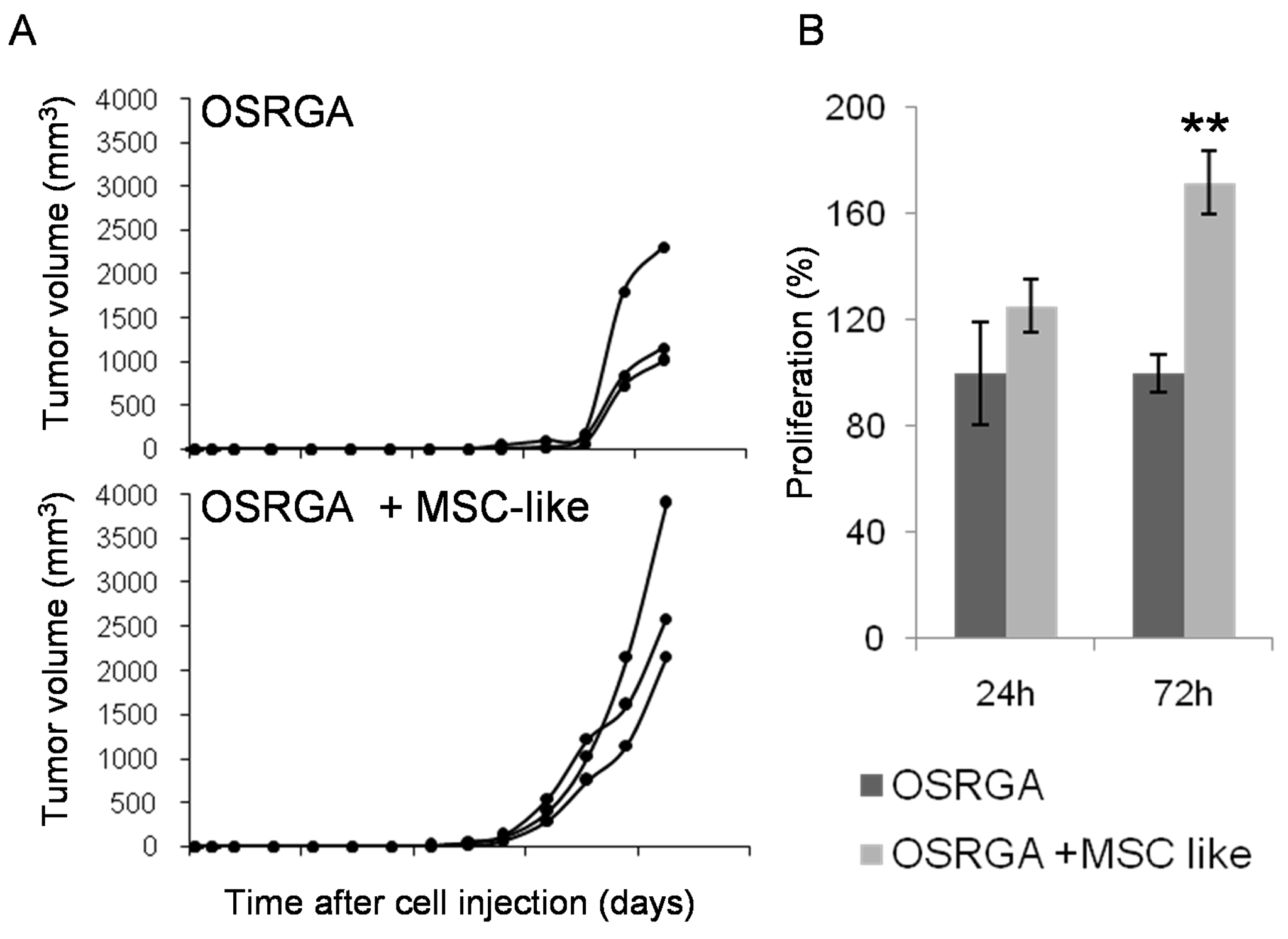

Supplement: Figure S1 — Rat MSCs interact with osteosarcoma cells. (A) Evolutions of the tumor volume induced into the footpad of nude mice (NMRI nu/nu; Elevages Janvier, Le Genest St Isle, France). The OSRGA cells were derived from a transplantable rat osteosarcoma model originally induced by radiation [11]. Relatively undifferentiated mesenchymal cells (MSC-like cells) were obtained from calvaria of newborn rat (2 days old Sprague-Dawley rat, Charles River, L'Arbresle, France) as previously described. Mice of the control group (OSRGA) received 106 OSRGA cells alone, while OSRGA cells were associated with calvaria-derived MSC-like cells at ratio 2∶1 in the OSRGA + MSC group. A third group received 0.5×10^6^ MSC-like cells alone and have not developed any tumor after 85 days (data not shown). The significance test is not performed as only three mice per group were included in this preliminary experiment. (B) OSRGA cells and calvaria-derived MSC-like cells were co-cultured without cell-cell contact. The OSRGA cell proliferation was analyzed by trypan blue cell counting after 24 and 72 hours. Results are presented as proliferation percentages relatively to the total number of OSRGA cells cultured alone. Error bars represent standard deviations and asterisks indicate significant differences between means (p<0.01). (0.50 MB TIF) [file pone.0010999.s001.tif]
